# miR-26a-5p/ADAM17-Mediated Proteolysis of TREM2 Regulates Neuroinflammation in Hypertensive Mice Following Lead Exposure

**DOI:** 10.3390/toxics13010037

**Published:** 2025-01-05

**Authors:** Yuran Wang, Zeming Wang, Han Hao, Yuwei Zhao, Jian Wang, Weixuan Wang

**Affiliations:** 1 School of Public Health, North China University of Science and Technology, Tangshan 063210, China; chyna_wang@163.com (Y.W.); wangzm@stu.ncst.edu.cn (Z.W.); haohan@stu.ncst.edu.cn (H.H.); yuweizhao@stu.ncst.edu.cn (Y.Z.); 2 Hebei Key Laboratory of Occupational Health and Safety for Coal Industry, Tangshan 063210, China; 3 The Laboratory Animal Center, North China University of Science and Technology, Tangshan 063210, China; wangjian_ncst@163.com

**Keywords:** hypertension, Pb exposure, miR-26a-5p, ADAM17, microglia, TREM2, neuroinflammation

## Abstract

Hypertension is not merely a vascular disorder but a significant risk factor for neural impairment. Moreover, healthcare for the hypertensive population with environmental or occupational pollutants has become an issue of increasing concern in public health. As a traditional neurotoxic heavy metal, Pb exposure results in neuroinflammation as well as neurodegenerative diseases. The current study aimed to investigate the mechanisms of neuroinflammation in hypertensive mice exposed to Pb. We demonstrated that hypertension exacerbated Pb-induced neuroinflammation in the prefrontal cortex (PFC), hippocampus, and hypothalamus, as evidenced by increased levels of proinflammatory cytokines (IL-6 and TNF-α) and decreased levels of anti-inflammatory cytokines (CD206 and IL-10). Additionally, hypertension enhanced the neuroinflammatory response in microglia, as indicated by similar changes in cytokine expression in an in vitro cell model. Importantly, we found that TREM2, a key regulator of microglial inflammation, was downregulated in hypertensive mice with Pb exposure. This decline in TREM2 expression was associated with increased proteolysis of TREM2 by a disintegrin and metalloproteases 10 (ADAM10) as well as a disintegrin and metalloproteases 17 (ADAM17), in which ADAM17 was verified as the main cleavage enzyme in terms of TREM2 proteolytic cleavage in hypertensive mice following Pb exposure. Furthermore, we identified miR-26a-5p as a potential regulator of ADAM17 expression, suggesting a potential mechanism for the downregulation of TREM2 in this context. Our findings provided new insights into the complex interplay between hypertension, Pb exposure, and neuroinflammation as well as highlight the potential role of TREM2, ADAM17, and miR-26a-5p as therapeutic targets for neuroinflammation in hypertensive populations with Pb exposure.

## 1. Introduction

Hypertension is a major health concern globally that affects nearly 30% of the world’s population [1]. Hypertension is not merely a vascular disorder but also a significant risk factor for neural damage, contributing to the pathogenesis of several neurological conditions and cognitive dysfunctions [2]. The pathophysiological mechanisms underlying hypertension-induced neural damage are multifaceted, involving a complex interplay between systemic hemodynamic changes, endothelial dysfunction, oxidative stress, and consequent neuroinflammation [3]. Meanwhile, the hypertensive population with neurotoxicants, especially environmental pollutants, have become an issue of increasing concern in terms of neurotoxicity. As we know, Pb is a traditional neurotoxicant that exists widely in the living environment. It is reported that Pb exposure can result in memory loss and slowness of reaction by neuroinflammation [4]. However, the knowledge gaps remain regarding the mechanism of neuroinflammation in the hypertensive population following Pb exposure.

As we know, microglia play a crucial role in the inflammatory response as the first line of defense [5]. Microglia-associated neuroinflammation is vital in the development of neurodegenerative diseases, such as Alzheimer’s disease (AD) [6]. Moreover, anti-inflammatory effects are generally considered to be beneficial for the treatment of diseases; for example, the anti-inflammatory agents contribute to antidepressant treatment [7]. Furthermore, some strategies relate to neuroinflammation by enhancing anti-inflammatory actions; for example, the anti-inflammatory effect of AZD6244 was used to resist acrolein-induced neuroinflammation [8]. Nevertheless, the change in microglia-mediated inflammatory and anti-inflammatory responses in the hypertensive population following Pb exposure requires the further investigation.

The neuroinflammation of microglia is regulated by many proteins, among which the triggering receptor expressed on myeloid cells 2 (TREM2) is an important protein regulating microglial inflammation. TREM2 is a cell surface receptor mainly expressed in microglia of the CNS, and full-length TREM2 contains 230 amino acids [9]. TREM2 can regulate proinflammatory and anti-inflammatory responses, cytophagocytosis, energy metabolism, and so on [10]. Moreover, TREM2 is cleaved at the stalk region by a disintegrin and metalloproteases 10 (ADAM10) as well as a disintegrin and metalloproteases 17 (ADAM17), and then the soluble TREM2 (sTREM2) is produced [11]. Higher cerebrospinal fluid (CSF) sTREM2 levels was associated with greater cognitive decline at the preclinical AD stage [12]. Evidence from in vitro studies has accumulated to support the role of TREM2 in restricting inflammation and promoting phagocytosis [13]. However, whether TREM2 is involved in neuroinflammation in hypertensive mice after Pb exposure is still unknown.

ADAM10 and ADAM17 belong to a disintegrin and metalloprotease (ADAM) family, which is a type I transmembrane protein. One characteristic of the ADAM family is the proteolytic cleavage of certain proteins. However, whether ADAM10 or ADAM17 predominantly performs proteolysis in TREM2 following neuroinflammation in hypertensive mice after Pb exposure needs further clarification.

ADAM10 and ADAM17 can be regulated by miRNAs. MiR-365 could inhibit the migration of triple-negative breast cancer by targeting ADAM10 [14]. Studies have revealed that miR-449a could regulate the invasion of human non-small-cell lung carcinoma by targeting ADAM10 [15]. MiR-26a-5p could alleviate cardiac hypertrophy and dysfunction via targeting ADAM17 [16]. In addition, miR-145 alleviated sepsis-induced inflammatory responses and organ injury by targeting ADAM17 [17]. Nevertheless, miRNAs that can regulate ADAM10 or ADAM17 are still unknown in hypertensive mice after Pb exposure. Moreover, TarBase (version 9.0), miRDB (release 5.0), and TargetScan (version 8.0) are the effective tools to screen miRNAs that may regulate ADAM10 or ADAM17 in hypertensive mice with Pb exposure.

In this study, the atlas of TREM2 decline was investigated, and the relationship between TREM2 and microglia-mediated neuroinflammation was clarified in hypertensive mice after Pb exposure. Next, the main protease responsible for the reduction in TREM2 was elucidated in hypertensive mice after Pb exposure. At last, the miRNA that could regulate this protease in hypertensive mice after Pb exposure was screened. Our findings will provide the therapeutic target for neuroinflammation in the hypertensive population after Pb exposure.

## 2. Materials and Methods

### 2.1. Animal and Treatment

All C57BL/6 mice (8-week-old males) were purchased from Beijing HFK Bio Science Co., Ltd. (Beijing, China) [license no. SCXK (Jing) 2021–0010]. Mice were raised under specific pathogen-free conditions with a temperature of 22 °C and humidity of 60% in the laboratory animal center of North China University of Science and Technology. After one week of adaptive feeding, half of mice were given intraperitoneal injection of AngII (0.5 mg/kg/d, Cayman, 17150), with one week to establish the hypertension model, and mice with systolic blood pressure ≥140 mmHg were considered as the successful model of hypertensive mice [18]. All mice were randomly divided into control group, hypertension group, Pb group, and Pb + hypertension group, with 50 mice in each group. Mice in the control group were given 0.9% normal saline by intraperitoneal injection every two days and pure water to drink every day. The mice in the hypertension group and Pb + hypertension group were given an intraperitoneal injection of AngII (0.5 mg/kg/d) every two days to maintain hypertension. In addition, mice in the Pb group and the Pb + hypertension group were given drinking water with 250 mg/L lead acetate (Sigma, Camas, WA, USA), and at weeks 2, 4, 8, 12, and 24, 10 mice from each group were selected and anesthetized, and their brain tissues were separated.

Twenty 8-week-old male TREM2 KO mice (TREM2^−/−^) were purchased from Saiye Model Biological Research Center (Taicang) Co., Ltd. (Guangzhou, China). Half of mice were given intraperitoneal injection of AngII (0.5 mg/kg/d) for one week to establish the hypertension model. Then, regular mice and hypertension mice were randomly divided into the control or Pb + hypertension group, with 10 mice in each group. The control mice were treated with 0.9% normal saline by intraperitoneal injection every two days and drank pure water freely; Pb + hypertension group mice were treated with AngII (0.5 mg/kg/d) every two days and Pb acetate in drinking water at doses of 250 mg/L for 12 weeks. All mice had blood pressure measured by mouse tail blood pressure measuring instrument (Kent, Tustin, CA, USA) every week until end of exposure. All experiments had been approved by the Animal Ethics Committee of North China University of Science and Technology (LAEC-NCST-2020082).

### 2.2. Methods of CSF Extraction in Mice

The head of the mouse was fixed at approximately 120° from the body, and the subcutaneous tissue and muscle of the mouse were blunted with tweezers. The white dural membrane of the mouse was found, and the tube reached the cerebellar bulbar cistern. The mouse cerebrospinal fluid was slowly extracted, and the tube was withdrawn. All samples were stored at −80 °C.

### 2.3. Cells Culture and Treatment

BV-2 cells were cultured in DMEM medium (Gibco, Waltham, MA, USA) containing 10% fetal bovine serum in the incubator (Yamato, Tokyo, Japan) at 37 °C with 5% CO_2_. The BV-2 cells were treated with 10 μM PbAC or/and 100 nM AngII when the BV-2 cells grew to 70%. While conducting the inhibitor experiments, BV-2 cells were treated firstly with GI (Cayman, Ann Arbor, MI, USA, 28284) to inhibit ADAM10 or TAPI-0 (MCE, Monmouth Junction, NJ, USA, HY-118694) to inhibit ADAM17 for 2 h then treated with 10μM PbAC and 100 nM AngII for 24 h.

### 2.4. Quantitative Real-Time PCR (qPCR)

Total RNA was extracted from cells and tissues using Trizol reagent (Invitrogen, Waltham, MA, USA), and the concentration of the extracted RNA was determined using a NanoDrop ND-100 spectrophotometer (NanoDrop Technologies, Wilmington, DE, USA). Then, total RNA was reverse-transcribed into cDNA using SuperScript II reverse transcriptase. qRT-PCR was performed using SYBR GreenER qPCR Super-Mix Universal with β-actin as the internal control. The primer sequences are in Table 1. Additionally, miRNA expression was detected using the miDETECT A Track™ miRNA qRT-PCR kit (RiboBio, Guangzhou, China). The sequences of miR-26a-5p, miR-26b-5p, miR-26a-3p, miR-194a-5p, and U6 small nuclear RNA, which are patent protected, were also purchased from RiboBio (Guangzhou, China).

### 2.5. Western Blotting

Proteins were extracted from brain tissues and BV-2 cells by using a BCA protein assay kit (PC0020, Solarbio, Beijing, China). The protein concentrations were detected using a bicinchoninic acid assay (BCA) kit. Then, the proteins were separated by 10% SDS-PAGE gel electrophoresis and transferred from the gel to the PVDF membrane. The primary antibodies used in the experiments included TREM2 (Huaan Biotechnology, Hangzhou, China, ER1902-96), ADAM10 (Proteintech, Rosemont, IL, USA, 25900-1-AP), ADAM17 (Huaan Biotechnology, ET1703-06), CD206 (Huaan Biotechnology, Hangzhou, China, ET1702-04), IL-10 (Affinity, Milwaukee, WI, USA, #DF6894), TNF-α (Proteintech, Rosemont, IL, USA, 60291-1-Ig), IL-6 (Proteintech, Rosemont, IL, USA, 21865-1-AP), and β-actin (Proteintech, Rosemont, IL, USA, 66009-1-lg), which was selected as an internal control, overnight at 4 °C. After washing with phosphate-buffered saline (PBS) + Tween-20 (PBST) three times, the second antibody was added and incubated for 2 h. Finally, ECL method was used for visualizing protein bands, and image J was used for analyzing the density of protein bands.

### 2.6. Immunofluorescence Staining

Mice were anaesthetized and perfused with 0.9% saline and 4% paraformaldehyde. Then, the brains of mice were extracted and immersed in 4% paraformaldehyde for 24 h at 4 °C. After that, the brains were dehydrated in 15% and 30% sucrose solutions for 24 h at 4 °C. Next, brains were cut into 20 µm sections. These sections were rinsed with PBS and permeabilized with 0.4% Triton for 2 h then blocked with 10% fetal bovine serum for 30 min. Moreover, the sections were incubated at 4 °C with antibodies, including TREM2 (ABclonal, Woburn, MA, USA, #A10482) and Iba-1 (Santa Cruz Biotechnology, Santa Cruz, CA, USA, sc-32725). After that, the sections were incubated for 2 h with Alexa 488 (green)-conjugated anti-rabbit IgG and Alexa 594 (red)-conjugated anti-mouse IgG antibodies. Finally, the sections were observed under a fluorescence microscope (FV3000; Olympus, Tokyo, Japan).

### 2.7. Behavioural Test

#### 2.7.1. Sucrose Preference Test (SPT)

All animals were trained before the formal test. Each animal was exposed to two bottles (one was 1% sucrose water, another one was drinking water), and the position of the bottles was switched after 24 h. Formal test was performed and lasted 24 h. Water consumption was calculated by measuring the volume of water before and after the test. Sucrose water preference was used as the evaluation index, and sucrose water preference (%) = intake of sucrose water/(intake of sucrose water + intake drinking water) × 100%.

#### 2.7.2. Elevated Plus-Maze Test (EPM)

The elevated plus-maze (O′Hara and Co., Ltd., Tokyo, Japan) test was associated with anxiety-like behavior. The mice were placed in the central area of the cross maze facing the open arm. Time spent in the open arms and the closed arms as well as the number of open arm and closed arm entries were recorded within 5 min test periods. Proportion of times to enter the open arm (OE%) = number of open arm/(number of open arm + number of closed arm); proportion of duration to stay in open arm (OT%) = open arm time/(open arm time + closed arm time).

### 2.8. Transfection

BV-2 cells were transfected with TREM2-expressing vectors or TREM2 siRNA (RiboBio, Guangzhou, China) into BV-2 cells using HighGene (ABclonal, Woburn, MA, USA, RM09014) according to transfection instructions before 10 μM Pb and 100 nM AngII exposure. Moreover, transfection of BV-2 cells with ADAM17 siRNA (RiboBio, Guangzhou, China) or miR-26a-5p mimics (GeneAdv, Suzhou, China) was performed according to the manufacturer’s instructions before Pb and AngII treatment.

### 2.9. Enzyme-Linked Immunosorbent Assay (Elisa)

CSF from hypertensive mice after Pb exposure and BV-2 cells culture supernatants were collected after different treatments; the levels of sTREM2 (Jianglai, Hefei, China) were detected using ELISA kit according to the manufacturer’s manuals. The minimum detection limit is 0.1 pg/mL.

### 2.10. Statistical Analysis

All data are presented as mean ± standard deviation (SD). One-way analysis of variance (ANOVA) was utilized to compare differences among multiple groups, and pairwise comparisons between groups were performed using the LSD test. Pearson correlation test was used to analyze the correlation between two continuous variables. The data analysis was conducted using IBM SPSS Statistics 23.0 (SPSS Inc., Chicago, IL, USA). If *p*-values were less than or equal to 0.05, the differences were deemed to be statistically significant, and relevant information is indicated in the figure legends.

## 3. Results

### 3.1. Hypertension Aggravated Neuroinflammation in Mice Following Pb Exposure

**Figure 1:**
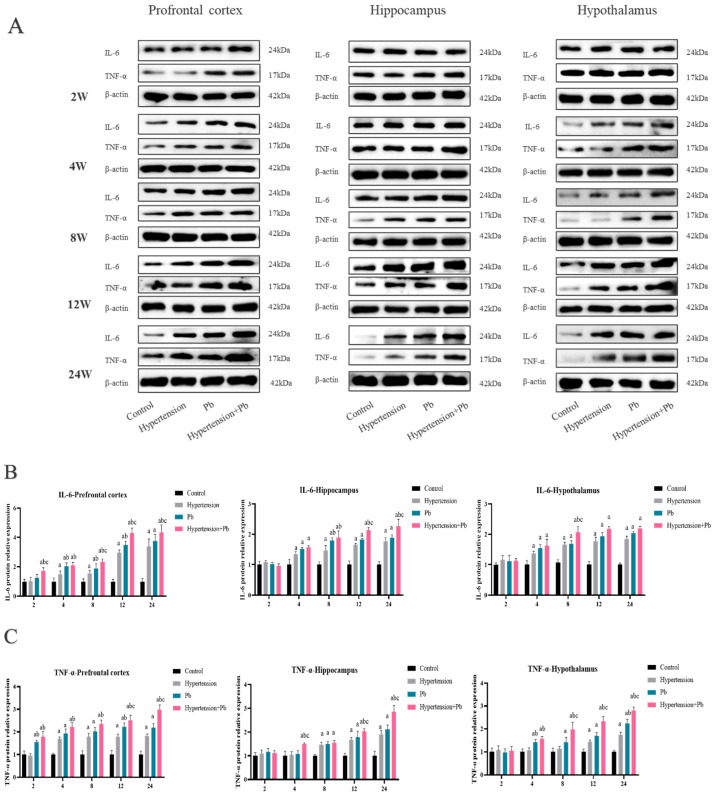

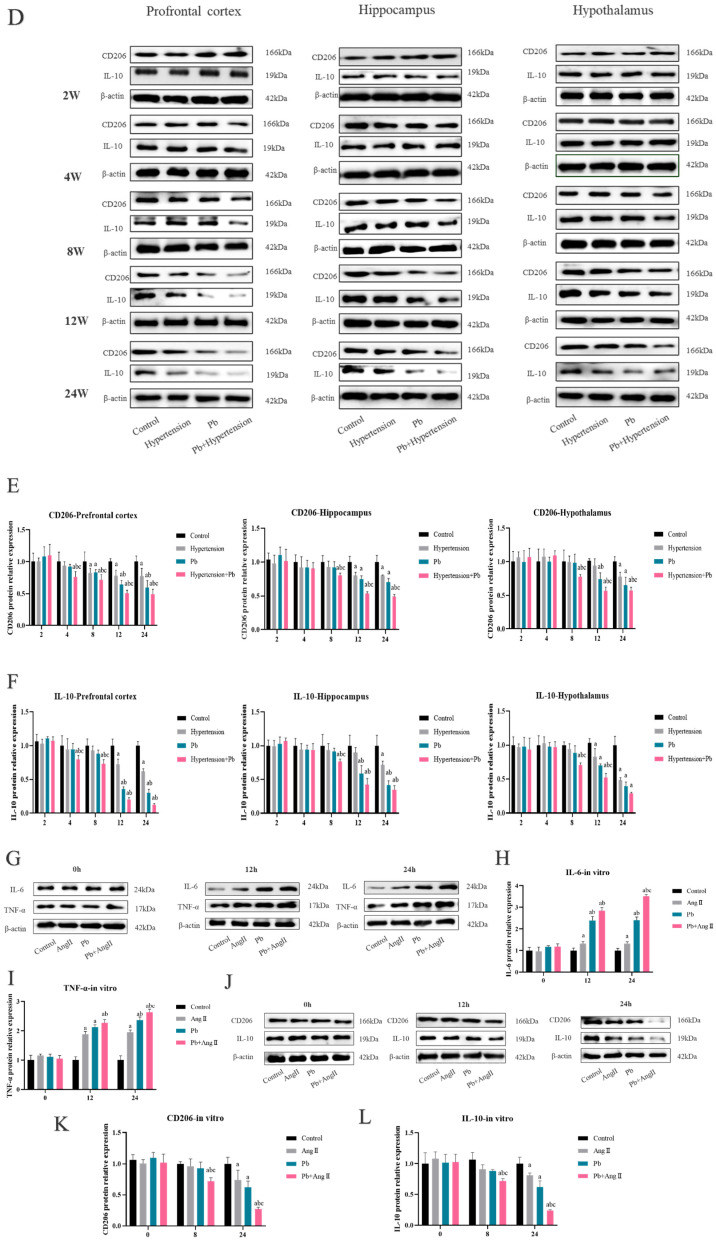
Hypertension aggravated neuroinflammation in mice following Pb exposure. (**A**) The change in protein expression levels of IL-6 and TNF-α in the PFC, hippocampus, and hypothalamus of hypertensive mice after Pb exposure. (**B**,**C**) Analysis of IL-6 and TNF-α protein expressions (*n* = 3). ^a^ *p* < 0.05 vs. control, ^b^ *p* < 0.05 vs. hypertension, ^c^ *p* < 0.05 vs. Pb. (**D**) The change in protein expression levels of CD206 and IL-10 in the PFC, hippocampus, and hypothalamus of hypertensive mice after Pb exposure. (**E**,**F**) Analysis of CD206 and IL-10 protein expressions (*n* = 3). ^a^ *p* < 0.05 vs. control, ^b^ *p* < 0.05 vs. hypertension, ^c^ *p* < 0.05 vs. Pb. (**G**,**J**) The change in protein expression levels of IL-6, TNF-α, CD206, and IL-10 in vitro following PbAc (10 μM) and AngII (100 nM) treatment at 0 h, 12 h, 24 h. (**H**,**I**,**K**,**L**) Analysis of IL-6, TNF-α, CD206, and IL-10 protein expressions (*n* = 3). ^a^ *p* < 0.05 vs. control, ^b^ *p* < 0.05 vs. AngII, ^c^ *p* < 0.05 vs. Pb.

To identify the inflammatory status along with Pb treatment, the protein expression of proinflammatory cytokines (IL-6 and TNF-α) in hypertensive mice after Pb exposure was detected in prefrontal cortex (PFC), hippocampus, and the hypothalamus of mice with/without hypertension and Pb exposure for 2 w, 4 w, 8 w, 12 w, and 24 w. The protein expression of IL-6 in the PFC of hypertensive or Pb-exposed mice gradually increased from 4 w and seemly peaked at 12 w exposure. Moreover, the IL-6 protein expression in the PFC of mice in the Pb + hypertension group heighted at 2 w exposure and reached to fourfold that of control group at 12 w exposure. Certainly, IL-6 expression in the hippocampus and hypothalamus enhanced from that at 4 w exposure in mice with hypertension or Pb exposure. In addition, hypertension resulted in higher TNF-α protein expression in the PFC, hippocampus, and hypothalamus of mice at 4 w, 8 w, and 12 w exposure, respectively. Moreover, Pb-exposed mice began to show higher TNF-α protein expression at 2 w exposure in the PFC and at 8 w exposure in the hippocampus and hypothalamus. As for the hypertensive mice with Pb exposure, TNF-α protein expression increased in the PFC, hippocampus, and hypothalamus at 2 w or 4 w exposure, respectively. Take together, hypertension could exacerbate the increase in IL-6 protein expression in the PFC of mice as early as 2 w into Pb exposure compared with that in the hippocampus or hypothalamus. In addition, hypertension significantly enhanced the expression of TNF-α of mice in the PFC, hippocampus, and hypothalamus after 12 w Pb exposure (Figure 1A–C).

Moreover, anti-inflammatory factors (CD206 and IL-10) were also detected. Among hypertensive mice, CD206 protein expression decreased in the PFC of mice at 8 w of treatment, following in the hippocampus and hypothalamus at 12 w and 24 w treatment, respectively. Meanwhile, CD206 protein expression in mice with Pb exposure is reduced in the PFC of mice at 8 w treatment, following in the hippocampus and hypothalamus at 12 w treatment. As we expected, CD206 expression in hypertensive mice with Pb exposure significantly decreased in the PFC at 4 w exposure, and following in the hippocampus and hypothalamus at 8 w treatment. In addition, IL-10 expression exhibited a comparable decrease in the PFC at 4 w, hippocampus and hypothalamus from 8 w of hypertensive mice with Pb exposure. The decrease in CD206 and IL-10 protein expression in the co-exposed group initially occurred in the PFC compared to that in the hippocampus and hypothalamus (Figure 1D–F). All those data indicated that hypertension might aggravate, especially in PFC, the decreased anti-inflammatory properties of mice with Pb exposure.

It is well-known that microglia sustain neuroinflammation; herein, the inflammation-related proteins including IL-6, TNF-α, CD206, and IL-10 were measured in BV-2 cells with Pb or AngII alone or combined at 0 h, 12 h, and 24 h with Pb or/and AngII treatment. As we expected, the protein expressions of IL-6, TNF-α, CD206, and IL-10 in BV2 cells showed similar changes in mice models (Figure 1G–L). The finding hinted that microglia-mediated neuroinflammation might play a pivotal role in hypertensive mice after Pb exposure.

In summary, the above data indicated that hypertension could enhance the neuroinflammation both in mice and microglia cells following Pb exposure.

### 3.2. The Decrease in TREM2 Expression in Microglia Was Accelerated by Co-Exposure to Pb and AngII

**Figure 2:**
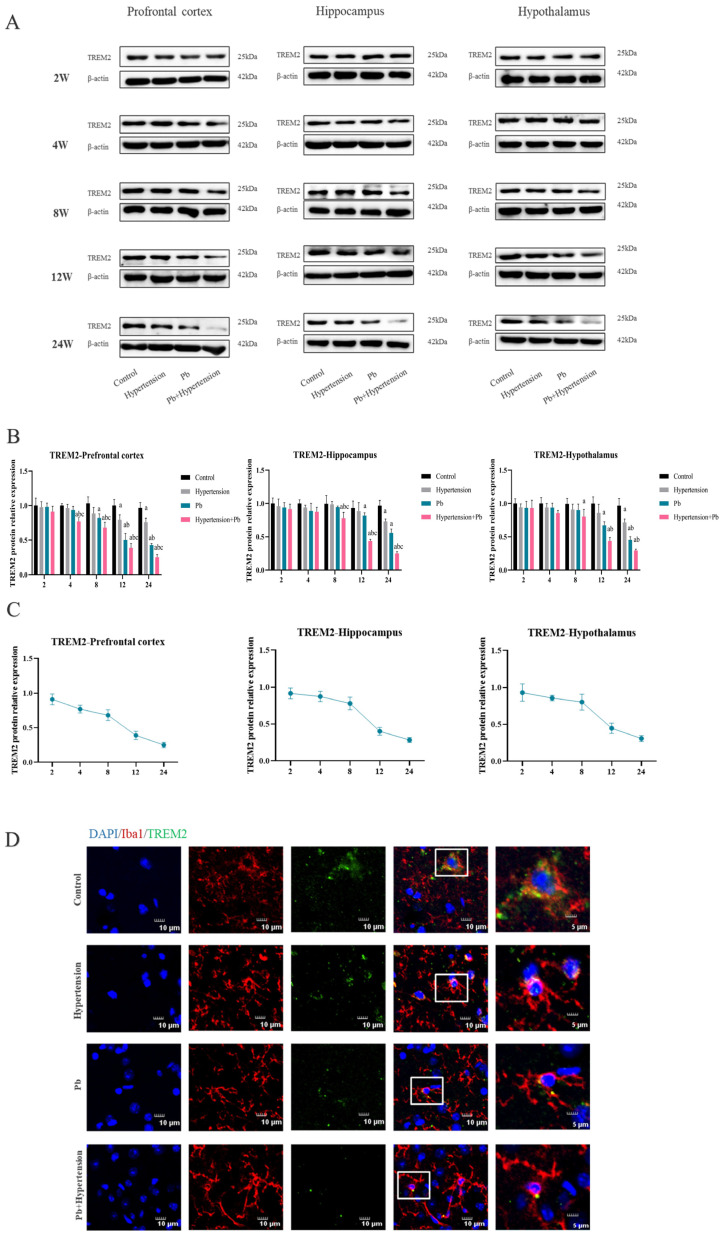

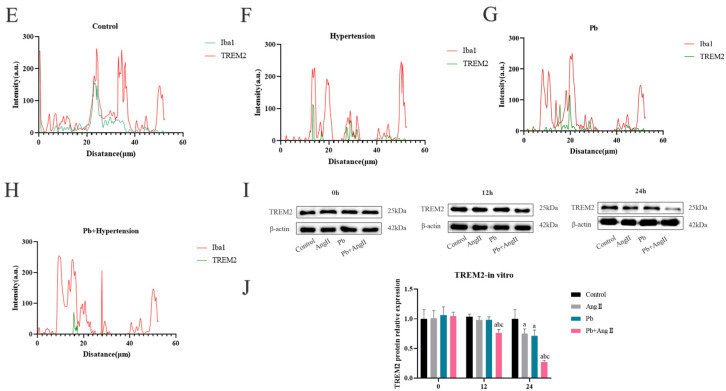
The decrease in TREM2 expression in microglia was accelerated by co-exposure to Pb and AngII. (**A**,**B**) The alteration and analysis of protein expression levels of TREM2 in the PFC, hippocampus, and hypothalamus in hypertensive or/and Pb-exposed mice (*n* = 3). ^a^ *p* < 0.05 vs. control, ^b^ *p* < 0.05 vs. hypertension, ^c^ *p* < 0.05 vs. Pb. (**C**) The trend of TREM2 protein expression of PFC, hippocampus, and hypothalamus in hypertensive mice after Pb exposure (*n* = 3). (**D**–**H**) Immunofluorescence images and analysis of TREM2 expression of microglia in the PFC. Red indicates Iba1 expression, green indicates TREM2 expression, and blue (DAPI) indicates the nucleus (*n* = 3). Square parts were zoomed in the last column, scale bar = 5 µm (**I**,**J**) The variation and analysis of protein expression levels of TREM2 in vitro following PbAc (10 μM) or/and AngII (100 nM) treatment at 0 h, 12 h, and 24 h (*n* = 3). ^a^ *p* < 0.05 vs. control, ^b^ *p* < 0.05 vs. AngII, ^c^ *p* < 0.05 vs. Pb.

Studies have revealed that TREM2 plays pivotal roles in regulating microglia-mediated neuroinflammation. To clarify the role of TREM2 expression herein, the atlas of changes in TREM2 protein expression was investigated in hypertension mice with Pb exposure. In the PFC, TREM2 protein expression of hypertensive and Pb-exposed mice decreased significantly from 12 w and 8 w exposure, respectively. Notably, hypertensive mice with Pb exposure had a lower expression of the TREM2 protein compared to the mice of hypertension or Pb alone from 4 w exposure and seemingly decreased significantly at 12 w exposure in which the protein expression of TREM2 was 38.4% that of control group. In the hippocampus and hypothalamus, TREM2 protein expression in the hypertensive mice and Pb-exposed mice descended from 24 w and 12 w exposure respectively, and the co-exposed mice began to reduce at 8 w treatment (Figure 2A,B). Essentially, the TREM2 protein expression reduced in hypertensive mice following Pb exposure. Moreover, the TREM2 protein expression of the co-exposed group decreased earlier than in the single-exposure groups and firstly appeared in the PFC. In addition to hypertension potentially exacerbating the decline of TREM2 in mice after Pb exposure, the TREM2 protein expression following co-exposure to hypertension and Pb showed the most significant decline speed at 12 w exposure in the PFC, hippocampus, and hypothalamus compared to that at other exposure time points (Figure 2C).

In order to further demonstrate the change in TREM2 expression that occurred in the microglia in hypertensive or/and Pb-exposed mice, the colocalization test of TREM2 and Iba1 was performed in the PFC of hypertensive mice following Pb exposure for 12 w. Hypertension and Pb exposure seemed to cause a decrease in the areas of cells with co-location of TREM2 and Iba1, and hypertensive mice with Pb exposure showed less than that of hypertensive mice or Pb-exposed mice (Figure 2D–H). Moreover, the BV-2 cells also revealed that the earliest and the most obvious change in TREM2 protein expression appeared in the Pb + AngII group (Figure 2I,J). Collectively, AngII treatment exacerbated the depletion of the TREM2 protein induced by Pb exposure.

### 3.3. TREM2 Played the Vital Role in Microglia-Related Neuroinflammation Caused by Pb and AngII Exposure

Given the relationship between TREM2 protein expression and neuroinflammation, we analyzed the association between TREM2 protein expression, proinflammatory factors (IL-6 and TNF-α), and anti-inflammatory cytokines (CD206 and IL-10). The results showed that there was strong negative correlation between TREM2 expression and IL-6, TNF-α expression but a strong positive correlation between TREM2 expression and CD206, IL-10 (Figure S1A,B).

**Figure 3:**
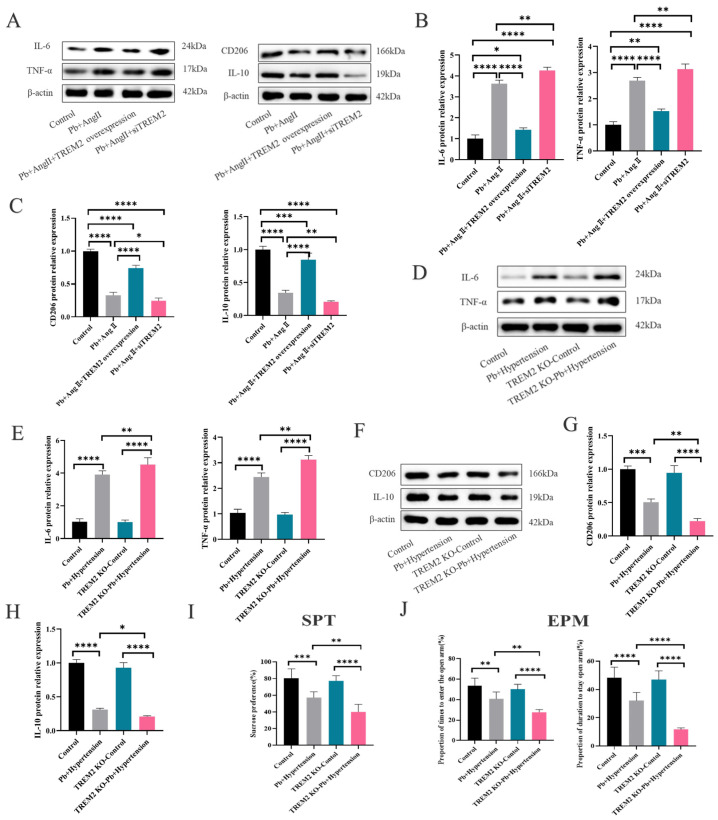
TREM2 played the vital role in microglia-related neuroinflammation caused by Pb and AngII exposure. (**A**–**C**) The protein expression levels and analysis of IL-6, TNF-α, CD206, IL-10 following Pb + AngII treatment after overexpressing TREM2 or knocking out TREM2 at 24 h in vitro (*n* = 3). **** *p* < 0.0001, *** *p* <0.001, ** *p* <0.01, * *p* < 0.05 vs. indicated group (The same below). (**D**–**H**) The protein expression and analysis of IL-6, TNF-α and CD206, IL-10 in C57 or TREM2 KO hypertensive mice after Pb exposure (*n* = 3). (**I**,**J**) The analysis of EPM, SPT (*n* = 10).

In order to figure out the role of TREM2 in regulating microglia-related neurotoxicity, we established the BV-2 cells with overexpressed or knockdown TREM2 (Figure S1C–H). Compared to BV-2 with Pb + AngII treatment, overexpressed TREM2 could increase the anti-inflammatory level (CD206, IL-10) and mitigate inflammatory level (IL-6, TNF-α) after Pb + AngII treatment, while knockdown TREM2 further exacerbated the anti-inflamatory expression (CD206, IL-10) and increased the inflammatory level (IL-6, TNF-α) following exposure to Pb + AngII (Figure 3A–C). In addition, we also applied TREM2 KO mice to further reveal the TREM2 value in terms of microglia-related inflammation. TREM2 KO hypertensive mice with Pb exposure exhibited further upregulated proinflammatory cytokines (IL-6, TNF-α) and downregulated anti-inflammatory comparing with C57 mice in the Pb + hypertension group (Figure 3D–H).

Moreover, the behavioral experiment also showed that the SPT of TREM2-KO hypertensive mice with Pb exposure were significantly lower than that in C57 hypertensive mice after Pb exposure. Subsequently, EPM had the similar tends such as sucrose preference test (Figure 3I,J). The dates indicated that the lack of TREM2 exacerbated anxiety and depression-like behaviors in the hypertensive mice after Pb exposure.

In a word, all above finding give solid evidence that TREM2 had a valuable role in regulating microglia-related neuroinflammation induced by Pb and AngII exposure.

### 3.4. The Change Profile of ADAM10 and ADAM17 in Hypertensive Mice with/Without Pb Exposure

**Figure 4:**
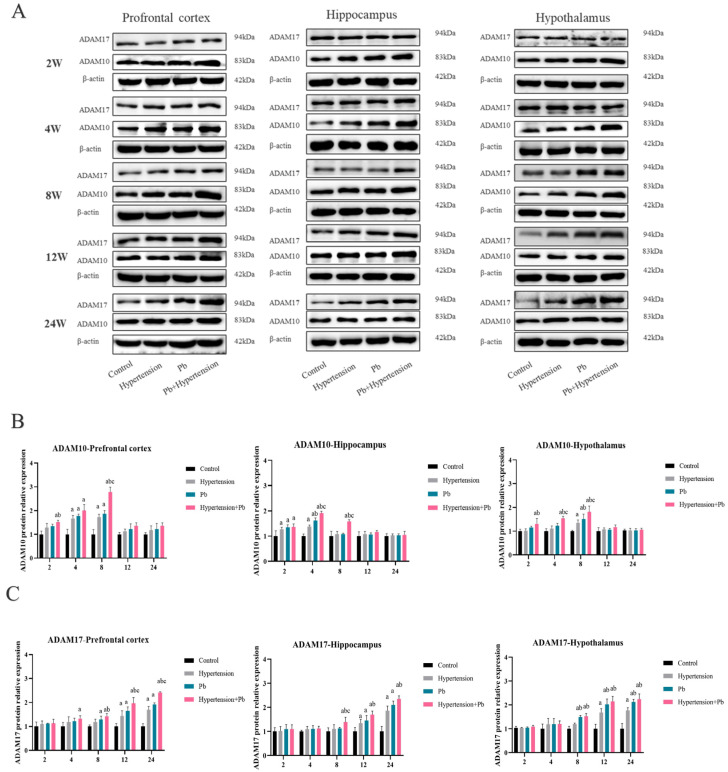
The change profiles of ADAM10 and ADAM17 in hypertensive mice with/without Pb exposure. (**A**) Protein expression levels of ADAM10 and ADAM17 in vivo. (**B**,**C**) The densitometric analysis of ADAM10 and ADAM17 in vivo (*n* = 3). ^a^ *p* < 0.05 vs. control, ^b^ *p* < 0.05 vs. hypertension, ^c^ *p* < 0.05 vs. Pb.

ADAM10 or ADAM17, the enzymes cleaving TREM2, were detected to elucidate the mechanism of TREM2 decrease in mice with hypertension, Pb alone, or co-exposure (Figure 4A–C).

ADAM10 protein expression in mice of the hypertension group or Pb group showed the upper in the hippocampus at 2 w exposure and in the PFC and hypothalamus at 4 w and 8 w exposure, respectively, peaking at 4 w or 8 w exposure. Meanwhile, whether hypertensive mice or Pb-exposed mice, ADAM10 protein expression was recovered to the control mice levels in hippocampus at 8 w and in PFC and hypothalamus at 12 w exposure. Certainly, the expression of ADAM10 in mice of the Pb + hypertension group rose from 2 w until peaking at 8 w in the PFC and hypothalamus, at 4 w exposure in the hippocampus; then, they returned to the control level after 12 w exposure. Simultaneously, the protein expression of ADAM17 in Pb-exposed mice began to increase in the PFC, in the hypothalamus at 8 w exposure, and in the hippocampus at 12 w exposure, and kept increasing trend until 24 w exposure in the PFC, hippocampus, and hypothalamus. Moreover, the ADAM17 protein expression of hypertensive mice began to enhance from 12 w exposure in three detected brain tissues, which were lagger than that of Pb exposure. Moreover, the ADAM17 protein expression of the Pb + hypertension group firstly increased in the PFC at 4 w exposure, following in the hippocampus and hypothalamus at 8 w exposure.

### 3.5. TREM2 Was Primarily Clipped by ADAM17 in Hypertensive Mice After Pb Exposure

**Figure 5:**
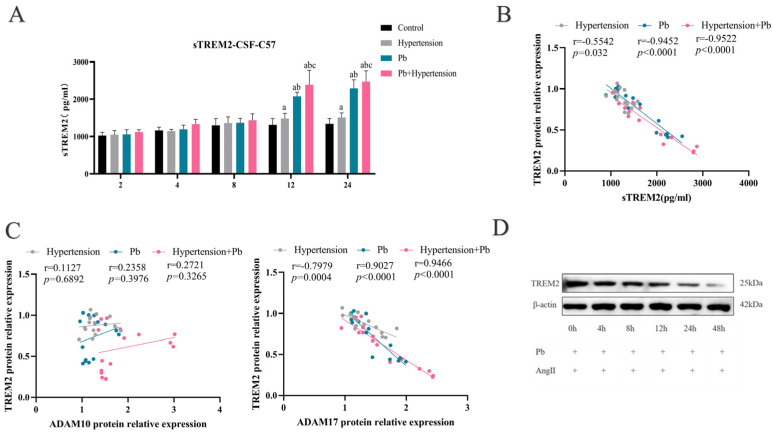

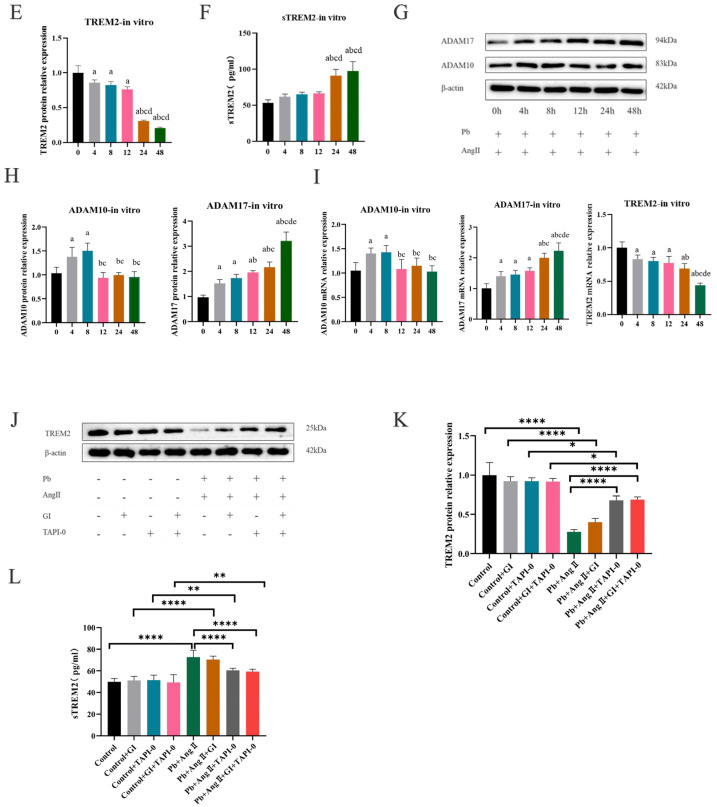
TREM2 was primarily clipped by ADAM17 in hypertensive mice after Pb exposure. (**A**) The alteration of sTREM2 in the CSF of hypertensive mice following Pb exposure (*n* = 6). ^a^ *p* < 0.05 vs. control, ^b^ *p* < 0.05 vs. hypertension, ^c^ *p* < 0.05 vs. Pb. (**B**) Association of TREM2 expression in the PFC of hypertensive and/or Pb-exposed mice with sTREM2 (*n* = 15). (**C**) Association of TREM2 expression in the PFC of hypertensive and/or Pb-exposed mice with ADAM10/17 (*n* = 15). (**D**–**I**) The change and analysis of protein expression of TREM2, ADAM10, and ADAM17 in BV-2 cells (*n* = 3). In addition, the expression of sTREM2, TREM2 mRNA, ADAM10 mRNA, and ADAM17 mRNA in BV-2 cells (*n* = 6). ^a^ *p* < 0.05 vs. 0 h, ^b^ *p* < 0.05 vs. 4 h, ^c^ *p* < 0.05 vs. 8 h, ^d^ *p* < 0.05 vs. 12 h, ^e^ *p* < 0.05 vs. 24 h. (**J**,**K**) Image and immunoblot analysis of TREM2 in BV-2 treated with 10μM TAPI-0 (ADAM17 inhibitor) or 20μM GI (ADAM10 inhibitor) following PbAc (10 μM) and AngII (100 nM) exposure (*n* = 3). (**L**) The expression of sTREM2 in BV-2 cells’ supernatant (*n* = 6). **** *p* < 0.0001, ** *p* <0.01, * *p* < 0.05 vs. indicated group.

It is reported that TREM2 can be sheared to sTREM2 by ADAM10 and ADAM17 [19], which might be one reason of decreased TREM2 in hypertensive mice with Pb exposure. Herein, we detected sTREM2 expression in the CSF of hypertensive mice with/without Pb exposure. The sTREM2 level in the CSF of the hypertension group, Pb group, and Pb + hypertension group significantly increased at 12 w and 24 w exposure, especially in the Pb + hypertension group (Figure 5A). However, the change in sTREM2 in the CSF lagged behind the change in TREM2 expression (Figure 2A and Figure 5A). Meanwhile, there was a strong negative correlation between CSF sTREM2 and TREM2 protein expression in the animal model (Figure 5B). Those data indicated that proteolytic enzyme (ADAM10/17) might be involved in TREM2 decline.

Then, the analysis showed that there was a negative correlation between TREM2 protein expression and ADAM17 protein expression in the PFC of mice (Figure 5C).

Next, we tested the protein expressions of TREM2 in BV-2 cells with Pb and AngII co-exposure in a time-response manner. A significantly decreased trend was observed from 4 h until 48 h treatment (Figure 5D,E). Moreover, the sTREM2 expression in BV-2 cells following Pb + AngII treatment demonstrated a gradual increase from 24 h Pb + AngII treatment (Figure 5F). Moreover, as depicted in Figure 5G,H, the ADAM17 protein expression increased in a time-dependent manner in response to Pb + AngII treatment. However, ADAM10 expression showed an increase at 2 h treatment that was back to the control levels at 12 h treatment. The relationship between TREM2 and ADAM17 displayed a negative correlation (Figure S2A). Simultaneously, the TREM2 and ADAM17 mRNA expressions showed comparable changes in protein expression (Figure 5I). All findings indicated ADAM17 might be a main proteolytic enzyme regarding the TREM2 decrease induced by Pb and AngII (hypertension) exposure.

To further clarify which served as the main proteolytic enzyme in terms of TREM2 degeneration, GI and TAPI-0 were applied for inhibiting ADAM10 and ADAM17, respectively (Figure 5J,K). Compared to the Pb + AngII group, the TREM2 protein expressions increased 2.43 folds in Pb + AngII + TAPI-0 group, but there was no significant change in the Pb + AngII + GI group. These data suggested that the decrease in TREM2 protein expression in microglia after Pb + AngII treatment was related to the high expression of ADAM17. Moreover, the sTREM2 expression also decreased, with TAPI-0 following Pb + AngII treatment as we expected (Figure 5L). In summary, ADAM17 was strongly associated with a decrease in TREM2 following Pb + AngII treatment.

Then, to further identify the role of ADAM17 in TREM2 after Pb and AngII treatment, we knocked down ADAM17 in BV-2 cells by siRNA with a decrease of 29.3% mRNA expression and 45% of the protein expression compared to the control (Figure S2B,C). The protein expression of TREM2 of an ADAM17^−/−^ BV-2 cell recovered to the control level of a cell with Pb + AngII treament (Figure S2D,E). Collectively, the results exhibited that ADAM17 is a major proteolytic enzyme of TREM2 regarding Pb and AngII (hypertension) treatment.

### 3.6. miR-26a-5p Regulated Pb- and AngII-Induced TREM2 Change by Targeting ADAM17

**Figure 6:**
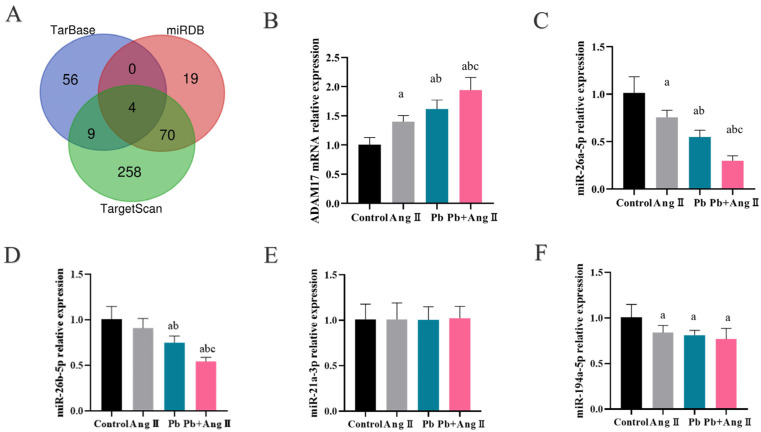

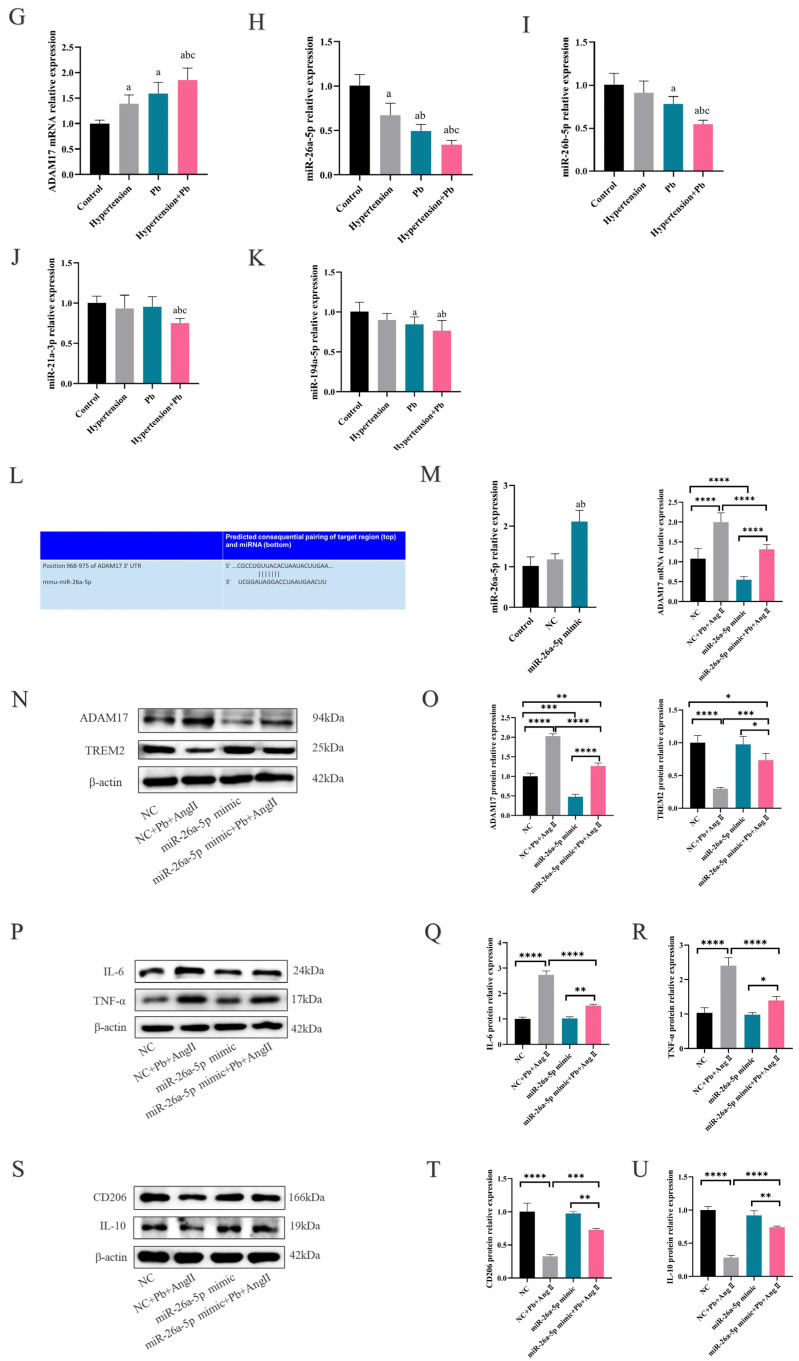
miR-26a-5p regulated Pb- and AngII-induced TREM2 change by targeting ADAM17. (**A**) Intersection of miRNAs that can regulate the ADAM17 by the databases that TarBase v.9.0, miRDB, and TargetScan 8.0. (**B**–**F**) The expressions of ADAM17 mRNA, miR-26a-5p, miR-26b-5p, miR-26a-3p, and miR-194a-5p in BV-2 treated by stimulation with PbAc (10 μm) or/and AngII (100 nM) for 24 h (n = 6). ^a^ *p* < 0.05 vs. control, ^b^ *p* < 0.05 vs. AngII, ^c^ *p* < 0.05 vs. Pb. (**G**–**K**) The expressions of ADAM17 mRNA, miR-26a-5p, miR-26b-5p, miR-26a-3p, and miR-194a-5p in the PFC following 12 w hypertension or/and Pb exposure (n = 6). ^a^ *p* < 0.05 vs. control, ^b^ *p* < 0.05 vs. hypertension, ^c^ *p* < 0.05 vs. Pb. (**L**) The binding site between miR-26a-5p and ADAM17 by TargetScan (version 8.0). (**M**) The expression of miR-26a-5p and ADAM17 mRNA in BV-2 (n = 6). ^a^ *p* < 0.05 vs. control, ^b^ *p* < 0.05 vs. NC. (**N**–**U**) The protein expression and analysis of ADAM17, TREM2, IL-6, TNF-α, CD206, and IL-10 with/without overexpressing miR-26a-5p in BV-2 following Pb (10 μm) and AngII (100 nM) for 24 h (n = 3). **** *p* < 0.0001, *** *p* <0.001, ** *p* <0.01, * *p* < 0.05 vs. indicated group.

The ADAM17 mRNA expressions of BV-2 cells in AngII or Pb treatment were higher than that of control group, and ADAM17 mRNA expression in the Pb + AngII group was higher than that of the Pb group and AngII group (Figure 6B). Meanwhile, the mRNA level of ADAM17 in the PFC at 12 w also increased, which was similar to the results in BV-2 cells (Figure 6G). To further elucidate the mechanism of ADAM17 elevation after AngII and Pb treatment, we applied TarBase v.9.0, miRDB, and TargetScan 8.0 to screen four miRNAs that regulated ADAM17, which were miR-26a-5p, miR-26b-5p, miR-26a-3p, and miR-194a-5p (Figure 6A).

Moreover, the expression of miR-26a-3p in the Pb + AngII group was the lowest compared to the control group in BV-2 cells, among miR-26a-3p, miR-26b-5p, miR-26a-3p, and miR-194a-5p (Figure 6C–F). The data showed that miR-26a-5p may regulate ADAM17 following Pb + AngII treatment.

The expression of miR-26a-3p, which was also lower than the other three miRNAs in the PFC with Pb + hypertension exposure, increased by 33% compared to the control group of hypertensive mice following 12 w PbAC exposure (Figure 6H–K).

It is reported that miR-26a-5p has a binding site with ADAM17 [20]; we overexpressed miR-26a-5p in BV-2 cells following Pb + AngII treatment, and ADAM17 mRNA expression was reduced by 31.57% that of Pb + AngII treatment and 63% for ADAM17 protein expression (Figure 6M–O). Meanwhile, TREM2 protein expression increased 1.5-fold compared to the Pb + AngII group (Figure 6N,O). Simultaneously, the IL-6 and TNF-α protein expression levels were decreased due to overexpressing miR-26a-5p (Figure 6P–R). In addition, the CD206, IL-10 protein expression levels in BV-2 treated for 24 h with Pb and AngII was improved after overexpressing miR-26a-5p (Figure 6S–U). Thus, the data above showed that miR-26a-5p could resolve the TREM2 decrease by regulating ADAM17 and further ameliorated TREM2-mediated inflammatory change.

## 4. Discussion

Pb exposure in the brain can result in a variety of neurological disorders, such as mental retardation, nerve damage, and possibly AD, PD, and so on [21]. However, the mechanism of neurological damage in the particular population of hypertension after Pb exposure is unclear. Microglia are considered the main immune cells in the CNS, and they respond when nerve damage occurs [22]. Microglia play major roles in responding to changes in the microenvironment with proinflammatory and anti-inflammatory responses to regulate the inflammatory process in the CNS [23]. However, the proinflammatory and anti-inflammatory level change in the microglia of hypertensive mice after Pb exposure is unclear. Recent studies have shown that IL-6 and TNF-α, which are common proinflammatory factors, could respond to inflammatory status. CD206 is highly expressed on microglia with an anti-inflammatory phenotype, and interleukin-10 (IL-10), a well-known anti-inflammatory cytokine, plays a key role in restricting inflammation [24]. To clarify microglia proinflammatory and anti-inflammatory responses in hypertensive mice after Pb exposure, we detected the protein expression of proinflammatory cytokines (IL-6 and TNF-α) and anti-inflammatory factors (CD206 and IL-10). Biochemical analysis revealed that hypertension aggravated proinflammatory levels’ increase and anti-inflammatory expressions’ decline in mice exposed to Pb, corresponding in cell model results (Figure 1). In particular, the results showed that the inflammatory change occurred earliest in the PFC. The PFC may be a sensitive brain region of neuroinflammation in hypertensive mice after Pb exposure.

In addition, TREM2 played an increasingly important role of regulating microglia changes in recent years [25,26]. TREM2 has been proved to exert a pivotal role in regulating neuroinflammation and is main expressed on microglia [27]. To clarify the effect of TREM2 in hypertensive mice following Pb exposure, firstly, we investigated the protein expression level of TREM2 in vivo and in vitro after exposure (Figure 2). The results in vivo showed that the expression of TREM2 protein of the PFC in hypertensive mice after Pb exposure decreased at 4 w exposure, while the expression of the TREM2 protein in the hippocampus and hypothalamus decreased at 8 w, with both of them only exerted in the Pb + hypertension group. These data suggested that hypertension could aggravate the decrease in TREM2 protein expression in the brain in mice exposed to Pb. Moreover, to further elucidate the role of TREM2 in hypertensive mice after Pb exposure, we carried out overexpressed and knockout TREM2 on the microglia (Figure 2). The results showed that Pb- and AngII-mediated inflammatory expression levels could be resolved by overexpressing TREM2. Our current results were consistent with TREM2 overexpression mitigating Pb-mediated inhibition of anti-inflammatory functions and alleviating Pb-induced microglial inflammation [28]. Moreover, TREM2 KO mice following hypertension and Pb exposure further aggravated inflammatory levels and anxiety–depression-like behavior.

The proteolytic shedding of TREM2 by ADAM10 and ADAM17 can result in the release of sTREM2 [29,30]. To further clarify whether the decline in TREM2 was associated with ADAM10/17, we firstly examined sTREM2 expression in the CSF of hypertensive or/and Pb-exposed mice. We found a time lag between the changes in sTREM2 and TREM2 had, with the alteration of sTREM2 being later than that of TREM2. Moreover, the analysis showed that TREM2 protein expression had a strongly negative correlation with sTREM2. Next, to further elucidate whether ADAM10 or ADAM17 were mainly involved in the proteolytic shedding of TREM2 following hypertension and Pb exposure, we examined the protein expressions of ADAM10 and ADAM17 in vivo (Figure 3). We found the TREM2 expression decline following Pb + AngII treatment to be related to the high expression of ADAM17. Moreover, BV-2 cells were pretreated with GI to inhibit ADAM10 or TAPI-0 to inhibit ADAM17 following Pb + AngII treatment, which finally demonstrated that ADAM17 mainly led to TREM2 decrease following Pb + AngII exposure.

Based on results above, the main proteolytic cleavage of TREM2 was ADAM17 in hypertensive mice after Pb exposure. More and more studies in recent years have shown that many miRNAs play an important role in neural injury recovery [31]. MicroRNAs (miRNAs) are a class of endogenous small RNAs with a length of about 20–24 nucleotides that can regulate many biological processes, such as cell proliferation, differentiation, and apoptosis by binding to the 3′-untranslated region (3′-UTR) of target messenger RNAs (mRNAs), resulting in gene silence [32]. Furthermore, miRNAs have an impact on key processes such as extracellular axon growth and intracellular signal transduction, and they also play a synergistic role in molecular pathways such as inflammation and apoptosis by regulating specific mRNAs and proteins [33]. Therefore, to further elucidate the reason of ADAM17 elevation in hypertensive mice after Pb exposure, we used TarBase v.9.0, miRDB, and TargetScan 8.0 to find the upstream regulatory target of ADAM17 and screened out four miRNAs, which were miR-26a-5p, miR-26b-5p, miR-26a-3p, and miR-194a-5p (Figure 4). According to our results, miR-26a-5p also played an important role in neuroinflammation, and overexpressing miR-26a-5p could resolve the TREM2 decrease by targeting ADAM17 and then alleviating the inflammatory levels in microglia following Pb and AngII treatment. In recent years, miR-26a-5p has shown an increasing effect in the CNS field [34]. In AD, overexpression of miR-26a-5p inhibited Tau phosphorylation and Aβ accumulation [35].

In summary, we used animal and cell models to explore the potential reason of microglia-mediated neuroinflammation after Pb and AngII treatment. The findings disclosed that hypertension significantly exacerbated inflammatory expression in Pb and AngII co-exposure compared to that in single exposure. TREM2, as a key regulator, played an important role in neuroinflammation in hypertensive mice after Pb exposure. Moreover, miR-26a-5p/ADAM17 can be used as a potential therapeutic target for TREM2 proteolytic cleavage following Pb and AngII exposure.

There are several limitations in this study. On the one hand, we only selected four cytokines (IL-6, TNF-α, CD206, and IL-10) as indicators of neuroinflammation. Actually, more cytokines should be selected to investigate the process of neuroinflammation to figure out which cytokine is sensitive for hypertensive mice following Pb exposure. On the other hand, the mechanism of the TREM2 decrease can be attributed to many pathways, such as mRNA regulation, protein autophagy, etc. Therefore, more experiments are needed to verify the role of other pathways on TREM2 decrease. Notably, sTREM2 levels were associated with greater cognitive decline at the preclinical AD stage and are more easily detected in serum, which could potentially serve as early diagnostic biomarkers for nerve injury in hypertensive individuals after exposure.

## 5. Conclusions

The study exhibits that miR-26a-5p/ADAM17 can cleave TREM2, alleviating microglia-mediated neuroinflammation in hypertensive mice following Pb exposure. MiR-26a-5p can be used as a potential therapeutic target to relieve microglia-mediated neuroinflammation in hypertensive people after Pb exposure.

## Figures and Tables

**Table 1:** Primer sequences.

Gene	Forward Primer (5′ to 3′)	Reverse Primer (5′ to 3′)
TREM2	TCTGAAGAACCTCCAAGCCG	GGAGGTGCTGTGTTCCACTT
ADAM10	TGAAGTGGAGCGAGAGGGAG	GTGCATCGATCCTGAGGGAG
ADAM17	AGCTGCCAAGTCCTTTGAGG	TGCTTCCCCGTTTCTCAGAT
β-actin	CATTGCTGACAGGATGCAGAAGG	TGCTGGAAGGTGGACAGTGAGG

## Data Availability

Data will be made available on request.

## Supplementary Materials

The following supporting information can be downloaded at: https://www.mdpi.com/article/10.3390/toxics13010037/s1, Figure S1: TREM2 played the vital role in microglia related neuroinflammation caused by Pb and AngII exposure. Figure S2: TREM2 was primarily clipped by ADAM17 in hypertensive mice after Pb exposure.
