# Supplementary material for: miR-26a-5p/ADAM17-Mediated Proteolysis of TREM2 Regulates Neuroinflammation in Hypertensive Mice Following Lead Exposure
Source: Toxics. 2025 Jan 5;13(1):37. doi: 10.3390/toxics13010037 (PMC11769360; doi:10.3390/toxics13010037)
Supplement: Supplementary file 1 [file toxics-13-00037-s001.zip › toxics-3400852-supplementary.pdf]

Supplementary material-figures

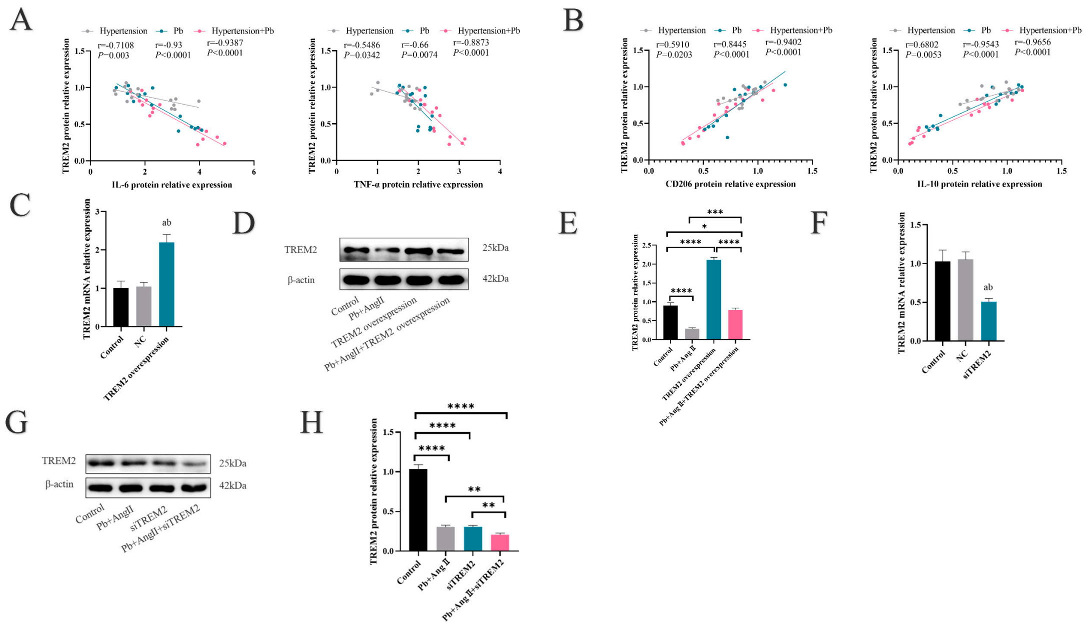

Figure S1. TREM2 played the vital role in microglia related neuroinflammation caused by Pb and AngII exposure.

(A) Association of TREM2 expression in the PFC of hypertensive and/or Pb-exposed mice with IL-6, TNF-α (n=15).

(B) Association of TREM2 expression in the PFC of hypertensive and/or Pb-exposed mice with CD206, IL-10 (n=15).

(C) The mRNA expression with/without TREM2 overexpression (n=6). $^{a}P < 0.05$ vs control, $^{b}P < 0.05$ vs NC.

(D) The protein expression levels of TREM2 in response to Pb+AngII treatment at 24h with/without overexpressing TREM2.

(E) Analysis of TREM2 protein expression (n=3). ****$p < 0.0001$, ***$p < 0.001$, **$p < 0.01$, *$p < 0.05$ vs indicated group (The same below).

(F) The mRNA expression with/without TREM2 knockout (n=6). $^{a}P < 0.05$ vs control, $^{b}P < 0.05$ vs NC.

(G) The protein expression levels of TREM2 in response to Pb+AngII treatment at 24h with/without knocking out TREM2.

(H) Analysis of TREM2 protein expression (n=3).

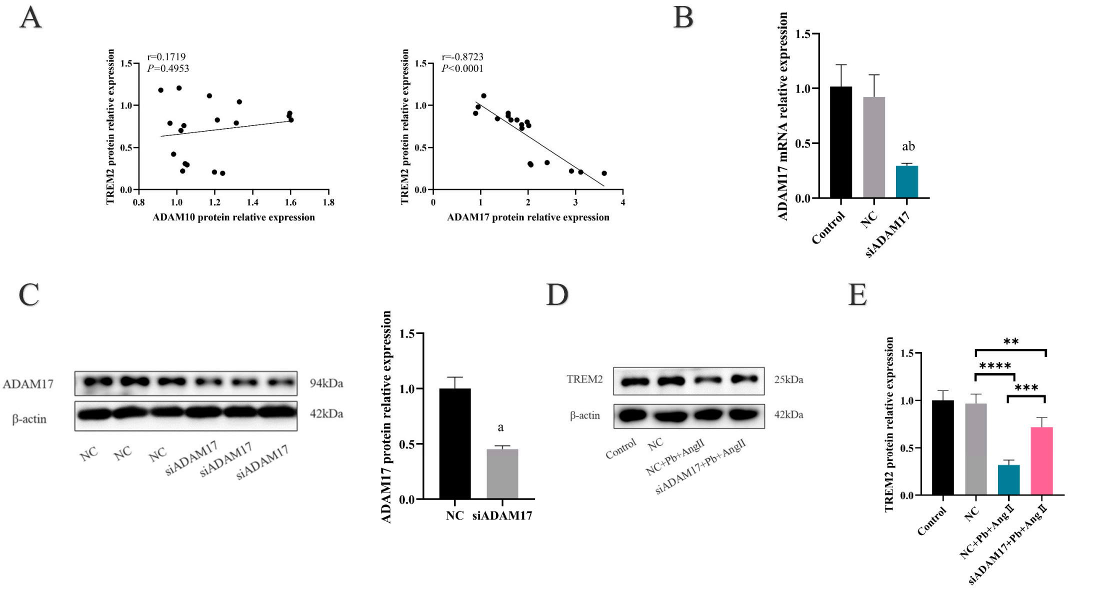

Figure S2. TREM2 was primarily clipped by ADAM17 in hypertensive mice after Pb exposure.

(A) Association of TREM2 expression in BV-2 cells of Pb+AngII treatment with the expression of ADAM10, ADAM17 (n=18).

(B) ADAM17 mRNA in BV-2 cells after siADAM17 transfection (n=6). $^{a}P < 0.05$ vs control, $^{b}P < 0.05$ vs NC.

(C) The image and statistical analysis of ADAM17 protein expression in BV-2 cells after siADAM17 transfection (n=3). $^{a}P < 0.05$ vs NC.

(D) The expression of TREM2 protein in BV-2 cells after siADAM17 transfection.

(E) The statistical analysis of TREM2 protein expression in BV-2 cells after siADAM17 transfection (n=3).
